# Patient perspectives on symptoms, health-related quality of life, and treatment experience associated with relapsed/refractory multiple myeloma

**DOI:** 10.1007/s00520-022-06979-7

**Published:** 2022-04-01

**Authors:** Nitya Nathwani, Jill Bell, Dasha Cherepanov, France Ginchereau Sowell, Rachel Shah, Kelly McCarrier, Parameswaran Hari

**Affiliations:** 1 Judy and Bernard Briskin Center for Multiple Myeloma Research, City of Hope Medical Center, Duarte, CA USA; 2 Takeda Development Center Americas, Inc (TDCA), Lexington, MA USA; 3 OPEN Health, Bethesda, MD USA; 4 Medical College of Wisconsin, Milwaukee, WI USA

**Keywords:** Patient-reported outcomes, Health-related quality of life, Patient experience, Treatment experience, Treatment burden, Relapsed refractory multiple myeloma

## Abstract

**Purpose:**

This study aimed to better understand the patient perspective and treatment experience of relapsed and/or refractory multiple myeloma (RRMM).

**Methods:**

This qualitative study enrolled adult RRMM patients from 6 US clinics who had ≥ 3 months of life expectancy, ≤ 6 prior lines of therapy, and ≥ 1 treatment regimen with a proteasome inhibitor and immunomodulator, or a CD38 monoclonal antibody or an alkylating agent, and a steroid. In-person semi-structured qualitative interviews were conducted to capture concepts that were relevant and important to patients. Topics included RRMM symptoms and impacts and the mode of administration, frequency, duration, convenience, side effects, and overall experience with RRMM treatment.

**Results:**

A total of 22 patients completed interviews. At enrollment, 59.1% of participants were using regimens containing dexamethasone, 36.4% daratumumab, 27.3% carfilzomib, and 18.2% lenalidomide. More participants had experience using intravenous or injectable therapy alone (40.9%) than oral therapy alone (18.2%). Back pain and fatigue were the most frequently reported symptoms (40.9% each); 27.3% reported no symptoms. Most participants reported physical function limitations (86.4%), emotional impacts (77.3%), MM-related activity limitations (72.7%), and sleep disturbances (63.6%). Most participants perceived treatment effectiveness based on physician-explained clinical signs (68.2%) and symptom relief (40.9%). Participants experienced gastrointestinal adverse events (59.1%), fatigue (59.1%), sleep disturbances (31.8%), and allergic reactions (31.8%) with treatment. Key elements of treatment burden included the duration of a typical treatment day (68.2%), treatment interfering with daily activities (54.5%), and infusion duration (50.0%).

**Conclusions:**

These results provide treatment experience–related data to further understand RRMM treatment burden and better inform treatment decision-making.

**Supplementary Information:**

The online version contains supplementary material available at 10.1007/s00520-022-06979-7.

## Introduction

Multiple myeloma (MM) is a clonal plasma cell malignancy with an incidence of 7.1 per 100,000 persons in the USA that was projected to account for an estimated 34,920 new cases (1.8% of all new cancer cases) and 12,410 deaths (2.0% of cancer deaths) in the year 2021 [1, 2]. It is the second most common hematological malignancy (10%) after non-Hodgkin lymphoma [3]. The typical age of onset in the USA is between 65 and 74 years [2], and the global incidence of MM is increasing [4], driven by factors such as the growing aging population, improved diagnostic capabilities, and increased awareness [5, 6]. Multiple myeloma is associated with significant morbidity and mortality characterized by impaired immune function, bone destruction, end-organ damage including renal failure, and death [4, 7].

Existing and emerging MM therapies, including proteasome inhibitors (PI), immunomodulators (IMiDs), and monoclonal antibodies, have dramatically improved median survival and 5-year survival rates in the past two decades [8–10]. Although the development of targeted therapies has improved treatment outcomes in patients with MM, most patients eventually relapse or become refractory to treatment [11]. Modern therapies have improved survival outcomes for patients with relapsed and/or refractory MM (RRMM), but prognosis is still poor [9, 12, 13]. Patients with RRMM require long-term treatment and an increased frequency of hospital and clinic visits, which is associated with indirect costs (e.g., time spent in hospital/clinic) and financial burden [14]. The increasing number of novel therapies and changing MM treatment goals have also increased the complexity of managing RRMM, as healthcare providers must consider patient-, disease-, and treatment-related factors as well as individual patient preferences when selecting treatments [11, 14–16].

More knowledge about patient experiences with treatments and their impact on everyday activities, including health-related quality of life (HRQOL), is needed for healthcare providers and patients to make informed treatment decisions [16–18]. This study aimed to gain a better understanding of the patient perspective on disease symptoms and impact and treatment experience with managing RRMM.

## Methods

### Study design and participants

This was a qualitative study of adult patients (≥ 18 years) in the USA with RRMM. Eligible patients had at least 1 prior MM relapse, a physician-confirmed life expectancy of at least 3 months, and were not eligible for stem cell transplant at the time of enrollment. Patients were eligible to enroll with up to 6 lines of prior therapy; the initial protocol included patients with up to 3 lines of prior therapy, but a protocol amendment of up to 6 lines was approved by the institutional review board (IRB) to facilitate broader patient recruitment. Eligible patients had received at least 1 regimen with a PI and IMiD, or a CD38 monoclonal antibody or an alkylating agent, and a steroid. Patients were also required to be able to read, write, speak, and understand English. Patients who had received stem cell transplant following relapse, had been diagnosed or treated for another malignancy within 2 years before MM diagnosis, or had any evidence of residual disease from a previously diagnosed malignancy were excluded. Patients were also excluded if they were diagnosed with a clinically relevant medical or psychiatric condition that could interfere with completing the study or had an uncontrolled/active infection or concurrent medical condition with symptoms that could confound their description of their experience with RRMM.

Purposeful sampling was used to enroll patients with RRMM from 6 community-based oncology clinics located in New York, Ohio, California, and Maryland. The participating sites were selected based on their expected ability to recruit eligible patients within the needed timeframe. Sites identified potential patients by screening medical records and databases to abstract key variables such as disease background and current treatment regimen. Potentially eligible patients were then contacted by phone or in person by site personnel using a prepared script summarizing the goals of the study and inviting them to participate in a one-time face-to-face interview. If the patient was interested, the site began the informed consent process. All participants provided written informed consent and agreed to the audio-recording and transcription of the interview. The site staff then abstracted additional descriptive demographic data about participants from the patient medical records. The study protocol was approved by Advarra IRB (Columbia, MD). Participants were compensated for time spent with a prepaid cash card presented in person at the end of the interview session.

### Qualitative interviews

Prior to conducting the RRMM patient interviews, we completed a targeted literature review and formative/pilot discussions with MM expert clinicians (*n* = 5) and patients (*n* = 3) to identify a list of concepts used to describe disease- and treatment-related experiences (Supplemental Fig. S1). This preliminary conceptual framework was used to guide the development of the semi-structured qualitative interview guide used in the RRMM patient interviews.

Study participants with RRMM were scheduled for an in-person concept elicitation (CE) interview in a private and comfortable setting at the clinic site. Each interview lasted 60 to 75 min. The interviewer used a semi-structured interview guide with follow-up probes when needed to capture the concepts that were relevant and important from the patient perspective and to conceptualize the symptoms, impacts (including physical, emotional, sleep, social, and functioning in daily life), treatment experiences, and treatment burden of RRMM.

An iterative approach was used to conduct two sets of interviews. Version 1 of the interview guide included questions involving the symptoms and impact of the disease in addition to participants’ treatment experiences. The interviews started with questions about symptoms participants experienced prior to being diagnosed with RRMM; then asked about symptoms they experienced during treatment, including whether there were any changes in their pre-diagnosis symptoms after treatment was initiated; and finally asked about impacts and treatment burden. In the initial wave of interviews, the research team observed patients’ emphasis on the burden of treatment. Hence, in the next wave of qualitative interviews, the research team focused more deeply on evaluating the treatment burden and benefit concepts that are relevant to patients with RRMM. Version 2 of the interview guide used targeted questions regarding the mode of administration, the frequency and duration of treatment, the overall convenience of receiving MM treatment, and the side effects of treatments. Questions focused on the convenience of MM medications and possible interference with daily activities and other factors leading to a favorable or unfavorable experience with treatment. Specific questions about the participant’s overall experience with treatment regimens and a typical day of treatment were also included in the second version of the interview guide.

### Qualitative coding and analysis

Audio-recordings were transcribed verbatim and coded for qualitative content analysis using ATLAS.ti version 8.0 (ATLAS.ti Scientific Software Development GmbH; Berlin, Germany). The goal of the coding process was to identify and organize relevant concepts and expressions of study participants related to the preliminary conceptual model and interview guide. Through the review of each verbatim transcript, specific codes were developed from the participants’ words, with conceptually equivalent codes grouped to represent distinct concepts within broad domains of RRMM experience (i.e., symptoms, HRQOL impacts) and treatment experience (i.e., effectiveness, tolerability, mode of administration). The preliminary coding scheme was refined until team consensus was reached, and consistency of coding was assessed through repeated consultations among coders. A subset of three transcripts was coded independently by 2 researchers; after the initial coding, coders met and compared the concepts identified and codes assigned, resolving any differences until a consensus was obtained. The coding then proceeded following the agreed upon coding scheme, and coders met regularly to discuss emerging themes and codes. Saturation of treatment experience concepts was assessed in the CE data through a process that examined the appearance of novel concepts across interview transcripts and in accordance with the order in which interviews were conducted.

## Results

### Participant characteristics

A total of 23 patients with RRMM enrolled in the study; of these, 22 participants completed qualitative interviews (one patient was excluded because they were unfit to participate at the time of their scheduled interview). Interviews were conducted from November 11, 2019, to February 25, 2020. The mean interview participant age was 69 years, and the majority of participants were male (Table 1). On average, participants had received their initial MM diagnosis 5.6 years (range: 1.6–12.8 years) prior to study enrollment. The mean number of relapses was 2.3 (range: 1.0–6.0), and most participants had ≤ 3 relapses (*n* = 18, 81.8%); the mean time between the most recent clinician-defined relapse and study enrollment was 1.4 years (range: 0–3.8 years). On average, participants had 3.3 comorbidities (range: 1.0–7.0). Hypertension (*n* = 9, 40.9%), respiratory illnesses (*n* = 7, 31.8%), and gastroesophageal reflux disease (*n* = 6, 27.3%) were among the most common comorbidities.

**Table 1:** Demographic and clinical characteristics at study enrollment^a^

Characteristics	RRMM patients (*N* = 22)
Age, mean (SD), years	68.9 (7.0)
Male sex, *n* (%)	13 (59.1)
Race, *n* (%)	
Black or African American	4 (18.2)
White	18 (81.8)
Education, n (%)	
High school education or less (≤ 12th grade/GED)	13 (59.0)
Further education (≥ 1–3 years of college)	8 (36.4)
Other^b^	1 (4.5)
Employment status, *n* (%)	
Employed/self-employed	3 (13.6)
Retired	12 (54.5)
Unemployed	1 (4.5)
Disabled	6 (27.3)
Time elapsed since MM diagnosis, mean (SD), years	5.6 (3.5)
Time elapsed since MM diagnosis, *n* (%)	
1–3 years	5 (22.7)
3–5 years	8 (36.4)
5–8 years	3 (13.6)
8–10 years	2 (9.1)
> 10 years	4 (18.2)
Time elapsed since most recent MM relapse, mean (SD), years	1.4 (1.1)
Number of relapses, *n* (%)	
1	7 (31.8)
2	8 (36.4)
3	3 (13.6)
≥ 4	4 (18.2)
Creatinine clearance, *n* (%)	
< 60 mL/min	7 (31.8)
≥ 60 mL/min	7 (31.8)
Not present in record^c^	8 (36.3)
Number of comorbidities, mean (SD)	3.3 (1.8)
Number of lines of therapy, mean (SD)	3.1 (1.2)
Type of current treatment regimen, *n* (%)	
No current treatment	2 (9.1)
Monotherapy	7 (31.8)
Doublet	7 (31.8)
Triplet	6 (27.3)
Current treatment mode of administration, *n* (%)	
Oral	4 (18.2)
Intravenous/Injectable	9 (40.9)
Both	9 (40.9)

Abbreviations: GED, general education development test; MM, multiple myeloma; RRMM, relapsed and/or refractory multiple myeloma.

^a^On average, patient interviews were completed 8 days after study enrollment. This gap ranged from same day to 22 days prior to interview (mean [range], 7.99 [0–22] days)

^b^Other education includes advanced training for work after 12th grade

^c^ One creatine clearance value was missing from data collection

Study participants had received a mean of 3.1 (range: 2.0–3.8) lines of therapy (Table 1). At enrollment, 59.1% of participants (*n* = 13) were using regimens containing dexamethasone, 36.4% (*n* = 8) daratumumab, 27.3% (*n* = 6) carfilzomib, and 18.2% (n = 4) lenalidomide; site staff reported all treatments included in the regimen, so treatments reported were not mutually exclusive. About one-third of patients used monotherapy (31.8%) or doublet therapy (31.8%) and 27.3% used triplet therapy. The mode of administration for participants’ current treatments varied: fewer participants were using oral therapy alone (*n* = 4, 18.2%) compared with intravenous or injectable treatment alone (*n* = 9, 40.9%) or both intravenous/injectable and oral treatment (*n* = 9, 40.9%).

#### Multiple myeloma experience

Example participant quotes regarding their MM experiences are shown in Table 2. Back pain (*n* = 9, 40.9%) and fatigue (*n* = 9, 40.9%) were the symptoms reported most frequently by participants; 27.3% of participants (*n* = 6) reported no symptoms (Table 3). Among the HRQOL impact concepts reported by participants (Table 4), physical function limitations (e.g., difficulty walking or standing) were most commonly reported (*n* = 19, 86.4%), followed by emotional impacts (e.g., worry/fear or sadness/depression) (*n* = 17, 77.3%), activity limitations related to MM (*n* = 16, 72.7%), and sleep disturbances (*n* = 14, 63.6%).

**Table 2:** Most frequently observed symptom and impact themes and select quotes based on semi-structured qualitative interviews with 22 RRMM participants

	No. (%) of patients expressing concept	Select quotes (patient characteristics)
**Symptoms**		
Back pain	9 (40.9)	*When I’m on Dexamethasone, I think the pain is less, everything works better…Saturday, Sunday it’s not good…It all returns.* (79 years, symptomatic, on fourth LOT after 3 relapses) *The treatment, really. I don’t ache as much. But I still hurt…My back…Yeah, but not as bad.* (76 years, symptomatic, on third LOT after 2 relapses)
Fatigue/tiredness	9 (40.9)	*Extreme tiredness…Yeah. Last, last month maybe not much of an appetite in the last month. That’s about added to the extreme fatigue, tiredness, like right now. Tired.* (80 years, symptomatic, on third LOT after 1 relapse) *But, I’m better now, I’m better, my energy level is better now than it was before I started my treatment.* (69 years, symptomatic, on second LOT after 2 relapses)
**HRQOL impacts**		
Activities limitations	16 (72.7)	*I can’t do the things I really want to. That really irritates me. I can’t do the things I used to do, but I’ve already prepared myself. I pace myself you know what I mean?* (70 years, symptomatic, on third LOT after 2 relapses)
Sleep problems	14 (63.6)	*Well, the, the steroids are, you know, it keeps you from sleeping at night.* (72 years, asymptomatic, on fourth LOT after 3 relapses) *It’s…Your, your energy level is up, you don’t sleep that night much, you’re wired, you can’t sleep, your brain just keeps going…* (69 years, symptomatic, on second LOT after 2 relapses)
Worry/fear	12 (54.5)	*Because your stomach feels like it’s swelling up and you can’t eat another drop and if you do eat, you can only eat a little bit at a time, so it’s things like that, and I don’t like to go anywhere, my kids get mad at me but I told me, I’d just rather be home and be comfortable than worrying about something happening anywhere else, you know.* (58 years, symptomatic, on second LOT after 1 relapse) *You know, in the beginning it was scary. But, you know, after a while you get used to it. You know what’s coming, it makes it a little easier, but uh, they’ve, I’ve been treated good. I’ve been treated good.* (68 years, asymptomatic, on fifth LOT after 5 relapses)

Abbreviation: HRQOL, health-related quality of life; LOT, line of therapy; RRMM, relapsed and/or refractory multiple myeloma.

**Table 3:** Frequency of symptom concepts reported by RRMM patients

Concepts	RRMM patients, *n* (%) (*N* = 22)
Pain	13 (59.1)
Back pain	9 (40.9)
Hip pain	3 (13.6)
Rib pain	2 (9.1)
Achiness	3 (13.6)
Jaw/tooth pain	3 (13.6)
Shoulder pain	2 (9.1)
Skin soreness	1 (4.5)
Leg pain	1 (4.5)
Neck pain	1 (4.5)
Arm pain	1 (4.5)
Hand pain	1 (4.5)
Fatigue/tiredness	9 (40.9)
Numbness/tingling	4 (18.2)
Weakness	3 (13.6)
Joint stiffness	1 (4.5)
Low appetite	1 (4.5)
Vomiting	1 (4.5)
Itchiness	1 (4.5)
No symptoms	6 (27.3)

Abbreviation: RRMM, relapsed and/or refractory multiple myeloma.

**Table 4:** Frequency of impact concepts reported by RRMM patients

Concepts	RRMM patients, *n* (%) (*N* = 22)
Physical functioning	19 (86.4)
Walking problems	9 (40.9)
Getting in/out bed or wheelchair	1 (4.5)
Lifting	1 (4.5)
Opening jars	1 (4.5)
Walking with support (walker)	1 (4.5)
Wheelchair	1 (4.5)
Going up/down steps	1 (4.5)
Standing/falling down	2 (9.1)
Emotional	17 (77.3)
Worry/fear	12 (54.5)
Sadness/depression	9 (40.9)
Disappointment/helplessness	6 (27.3)
Stigma/embarrassment	4 (18.2)
Others worry	2 (9.1)
Irritability	3 (13.6)
Feeling like burden to others	1 (4.5)
Activities limitations	16 (72.7)
Sleep problems	14 (63.6)
Social impacts	11 (50.0)
Require support from others	5 (22.7)
Limits playing with grandchildren	4 (18.2)
Caring for others	1 (4.5)
Does not want to socialize	2 (9.1)
Needing more rest	11 (50.0)
Activities of daily living	7 (31.8)
Difficulty cleaning/laundry	4 (18.2)
Difficulty mowing	2 (9.1)
Difficulty shoveling snow	1 (4.5)
Difficulty showering/bathing	1 (4.5)
Difficulty dressing	1 (4.5)
Difficulty cooking	1 (4.5)
Limitations when working	6 (27.3)
Limitations to driving	4 (18.2)
Weight fluctuations	3 (13.6)
Financial burden	3 (13.6)
Fracture	3 (13.6)
Not eating	3 (13.6)
Reading	1 (4.5)
Fainting	1 (4.5)

Abbreviation: RRMM, relapsed and/or refractory multiple myeloma.

### Treatment experience

Interview participants described their treatment experiences in three primary domains of interest: treatment effectiveness, treatment tolerability, and mode of administration (Table 5). Two effectiveness concepts were elicited during the interviews: symptom relief and clinical signs (Table 6). Ten RRMM participants (45.5%) reported that their treatment did not relieve their symptoms. Most participants (*n* = 15, 68.2%) perceived treatment effectiveness based on laboratory test results explained by their healthcare provider, and 40.9% (*n* = 9) based treatment effectiveness on symptom relief. Participants looked forward to receiving feedback about their laboratory results and not receiving it caused worry and concern. Without feedback, they were unable to tell whether the treatment was working or not and could not comment on the effectiveness of treatment with regards to clinical signs.

**Table 5:** Frequency of treatment experience concepts reported by RRMM patients

Concepts	RRMM patients, *n* (%) (*N* = 22)
**Treatment effectiveness**	
Symptom relief	22 (100.0)
Perceived effectiveness	9 (40.9)
Perceived lack of effectiveness	10 (45.5)
Unable to comment on effectiveness of treatment	4 (18.2)
Clinical signs	20 (90.9)
Perceived effectiveness	15 (68.2)
Perceived lack of effectiveness	12 (54.5)
Unable to comment on effectiveness of treatment	3 (13.6)
**Tolerability**	
Adverse events	19 (86.4)
Gastrointestinal symptoms	13 (59.1)
Fatigue	13 (59.1)
Sleep	7 (31.8)
Allergic reactions	7 (31.8)
Weakness	5 (22.7)
Alertness	4 (18.2)
Pain	3 (13.6)
Neurological symptoms	2 (9.1)
Breathlessness	2 (9.1)
Numbness	2 (9.1)
Stiffness	2 (9.1)
Swelling	2 (9.1)
Infections	2 (9.1)
Visual problems	2 (9.1)
Mood swings	2 (9.1)
Hair loss	2 (9.1)
Palpitations	1 (4.5)
Skin reactions	1 (4.5)
No side effects	8 (36.4)
**Mode of administration**	
Treatment regimen	22 (100.0)
Interference with regular activities	12 (54.5)
Treatment complexity	6 (27.3)
Distance to clinic	3 (13.6)
Treatment significance	2 (9.1)
Home versus clinic	1 (4.5)
Access to information	1 (4.5)
Fewer visits is sign of health	1 (4.5)
Typical day of treatment	22 (100.0)
Duration	15 (68.2)
Interference with activities	3 (13.6)
Fatigue	5 (22.7)
Setting	3 (13.6)
Pain	2 (9.1)
Waiting to receive treatment	3 (13.6)
Not receiving treatment	1 (4.5)
Infusion	21 (95.5)
Duration	11 (50.0)
Convenience	8 (36.4)
Effectiveness	5 (22.7)
Pain	7 (31.8)
Setting	4 (18.2)
Side effects	2 (9.1)
Nonissue	14 (63.6)
Injections	10 (45.5)
Duration	5 (22.7)
Pain	4 (18.2)
Effectiveness	1 (4.5)
Nonissue	3 (13.6)
Pills	10 (45.5)
Quantity	5 (22.7)
Convenience	4 (18.2)
Side effects	2 (9.1)
Adherence	1 (4.5)
Palatability	2 (9.1)
Effectiveness	1 (4.5)
Nonissue	1 (4.5)

Abbreviation: RRMM, relapsed and/or refractory multiple myeloma.

**Table 6:** Most frequently observed patient treatment experience themes and select quotes based on semi-structured qualitative interviews with 22 RRMM patients

	N (%) of patients expressing concept	Select quotes (patient characteristics)
**Treatment effectiveness**		
Symptom relief	22 (100.0)	*No, the pain is uh, more or less it took its course. It’s not bad, it’s not as bad as it was when I first got diagnosed. But this new chemo now it’s more pain reliever.* (70 years, symptomatic, on third LOT after 2 relapses) *I don’t know that I have any symptoms.* (72 years, asymptomatic, on fourth LOT after 3 relapses)
Clinical signs	20 (90.9)	*Well, I’m not sure of that. But, it brought my numbers down significantly lower than the previous medication.* (66 years, symptomatic, on second LOT after 2 relapses) *Well, I really don’t know when it starts working. It uh, it’s not that type of pill, you know, like where you have a headache and boom! You give a pill or something and it goes away. You know, after an hour or so. This is a long process. Right? … Well, I say when I come in till they check on my blood and find out that it’s low and then I have to check if they are going to give it to me, and if it was too low they won’t, and then to me that’s.. I wish they could get that part done faster, that they know whether I get the treatment or don’t get the treatment but otherwise…* (72 years, symptomatic, on fifth LOT after 4 relapses) *I think it’s helping, I think it’s helping because um, my only judge is those 24 hour urine tests, and looking at that urine protein. And if I go a month, take a 24 hour urine draw and that number is decreased, then I feel like I am gaining on the disease.* (79 years, symptomatic, on fourth LOT after 3 relapses)
**Treatment tolerability**		
Gastrointestinal adverse events	13 (59.1)	*I’m um, usually when I’m on my chemo, I’m tired, you know, I don’t, usually I don’t feel good I’m sort of nauseated on my stomach.* (66 years, symptomatic, on second LOT after 1 relapse)
Fatigue	13 (59.1)	*And [inaudible] and yeah, I was really tired before, so I’m tired now, probably from the side effects of the drugs maybe more so than the cancer.* (76 years, symptomatic, on third LOT after 2 relapses) *Usually twice, twice a week. Yeah, and usually the, after the second day of chemo, is when you know, I get the fatigue, it affects me more.* (71 years, symptomatic, on fourth LOT after 3 relapses)
**Modes of administration**		
Interference with daily activities	12 (54.5)	*You do have to plan your life around your treatments now, you know, if you want to go anywhere, you know, you have to make arrangements, or you know, just schedule around your treatments and so forth. People never think about that.* (69 years, symptomatic, on second LOT after 2 relapses)
Duration of typical day of treatment	15 (68.2)	*Sometimes the treatment was like so long, one thing after the other. Then, you know, but I’m glad it’s getting more simplified, that’s, yeah. You know, that’s what I see. That’s what I would say.* (80 years, symptomatic, on third LOT after 5 relapses)
Duration of infusion	11 (50.0)	*Um, yeah, because sometimes they had me on a machine…for a long time. Like for the whole day. Maybe for 8 hours or something like that. You know, that can kind of wear on you emotionally.* (80 years, symptomatic, on third LOT after 5 relapses) *About five hours. I usually finish by four o’clock…I don’t like it, not at all.* (76 years, symptomatic, on third LOT after 2 relapses)
Duration of injection	5 (22.7)	*Well, I like the injections in my arm. The Velcade and Xgeva are both injections, I get them in my arm, so that’s better than sitting there for 2 hours…infusion.* (58 years, symptomatic, on second LOT after 1 relapse)
Quantity of pills	5 (22.7)	*It was a lot. I think I had more and I’m forgetting what…like I had a whole bag of pills, much more than I ever had before. And I had to take them every single day. At one point I was taking so many pills I felt, I didn’t even want to take them anymore because looking at them, they’re huge, and it…it was like my goodness, do I need to take all of these?* (66 years, symptomatic, on second LOT after 2 relapses)

Abbreviation: RRMM, relapsed and/or refractory multiple myeloma.

A total of 18 treatment tolerability concepts were elicited during the interviews. Most participants described experiencing gastrointestinal adverse events (*n* = 13, 59.1%) and fatigue (*n* = 13, 59.1%). The next most commonly reported concepts were sleep disturbances (*n* = 7, 31.8%) and allergic reactions (*n* = 7, 31.8%). Eight participants (36.4%) reported no adverse events.

Regarding mode of administration concepts, participants most commonly reported the overall duration of a typical treatment day (*n* = 15, 68.2%), treatment interfering with daily activities (*n* = 12, 54.5%), and duration of infusion (*n* = 11, 50.0%) as key elements of treatment burden (Table 6). According to participants, receiving RRMM treatment involves scheduling activities around days of treatment, not taking trips, not feeling well enough to spend time with family members, and depending on others to rearrange their schedule to provide transportation for clinic visits. Several interview participants reported traveling up to 2 h to receive their infusion. Factors that make a typical treatment day longer included laboratory tests and analyses; the treatment itself, which can vary in length; and the addition of other treatments (e.g., blood transfusion). Participants reported being satisfied with more simplified days of treatments. Descriptions provided by participants suggested that long infusions are inconvenient, tiresome, and emotionally draining (Table 6). In contrast, participants reported being satisfied with shorter infusions. Compared to infusions, participants preferred the rapid administration of injections. For oral treatment, the most frequently reported concepts were quantity of pills (*n* = 5, 22.7%) and convenience (*n* = 4, 18.2%). Participants mentioned the physical and mental ease and time savings (e.g., less travel time) of taking pills as part of the convenience of oral treatment.

## Discussion

This qualitative study uncovered treatment experience-related concepts that study participants with RRMM reported as important. A central theme that resulted from this analysis was the burden of time dedicated to following the prescribed treatment regimen, with participant-perceived unfavorable factors including the duration of the typical day of treatment and the duration of infusions and injections of existing treatment. Participants indicated that they often plan their lives around treatment days due to the duration of infusion treatment and prolonged impacts of treatment-related adverse events that can occur. Many participants expressed experiencing gastrointestinal issues or fatigue and the need to plan their activities around when these adverse events have resolved and they have more energy.

Participants’ perceptions of treatment effectiveness depended largely on symptom relief and clinical signs. While many participants utilized symptom relief to indicate the effectiveness of the treatment, some had difficulty relying solely on this as they continued to experience symptoms following treatment and others were asymptomatic. Therefore, it is particularly important that healthcare providers share the results of the clinical tests with their patients, as many participants reported using this information to determine whether the treatment is working.

Participants in the first wave of interviews emphasized the burden of RRMM treatment, so we adjusted the interview guide for later waves to focus more on patient-reported treatment-related experiences than symptom-related concepts. However, the symptoms and impacts reported by study participants were consistent with the symptoms and impacts of RRMM reported in the literature. The adverse events endorsed by participants in this study were similar to those reported by participants with RRMM in the study conducted by Parsons et al. (e.g., fatigue, pain, gastrointestinal symptoms, sleep disturbances, mood swings) [18].

Our results were consistent with previous patient preference studies among patients with RRMM, in which drug administration (i.e., therapy mode of administration, number and duration of physician visits) has been identified as the most important factor in patients’ treatment decision-making [19, 20]. Mode of administration and the amount of time spent receiving therapy have also been identified as being associated with RRMM patients’ perceptions of treatment convenience [14]. In previous studies, some patients with RRMM have prioritized, preferred, and/or had higher satisfaction with oral treatments and in some cases have observed a willingness to accept a less effective therapy in exchange for more convenient treatments or treatments with less impact on quality of life (typically oral treatments) [19–21]. Compared with oral therapies, injectable therapies have been associated with increased time burden and higher indirect costs in patients with RRMM [14]. In the COLUMBA clinical trial, patients with RRMM reported greater treatment satisfaction with subcutaneous daratumumab therapy compared with intravenous infusion of daratumumab, and higher satisfaction with subcutaneous daratumumab therapy was maintained over 10 treatment cycles [22]. Previous research has shown that prolonged duration of therapy is associated with better outcomes in patients with RRMM and identified the need to remove barriers to the extended duration of treatment (i.e., until progression), such as improving treatment convenience [23]. Ensuring that RRMM treatment regimens are effective, tolerable, and convenient enough for patients may improve real-world adherence and enable patients to derive the full clinical benefits of treatment [16].

Previous qualitative studies have assessed the symptom burden of MM [24], so in light of participant responses, we shifted our focus in later interviews to treatment-related experiences rather than disease symptoms, as explained above. The treatment burden associated with the mode of administration for RRMM therapies is not typically examined in clinical trials, which focus more on safety and efficacy rather than other parts of the treatment experience that are important to patients (e.g., time, scheduling) [16]. Previous research has reported that the potential for treatment-free intervals is relevant to treatment decision-making for patients with MM as well as healthcare providers, suggesting that the burden of treatment plays a role in these treatment decisions and highlighting the need to gather evidence on patients’ treatment experiences [25]. Our qualitative interview results help fill the evidence gap for treatment experience–related data to further understand the patient experience as it relates to treatment burden in order to better inform treatment decision-making.

We acknowledge some limitations of our study. As is common among qualitative interview-based studies, the information reported here was drawn from a relatively small sample of participants (*N* = 22). Although evidence of concept saturation was observed in the qualitative dataset, suggesting the robustness of the qualitative analysis, caution should be taken when interpreting results due to the small sample size. The study protocol was amended partway through data collection and broadened the number of lines of prior therapy from up to 3 lines to up to 6 lines. The study population may be skewed toward patients with fewer relapses (68.2% of our study sample had ≤ 2 relapses); only four participants (18.2%) had ≥ 4 relapses, so we may have under-sampled patients with more substantial relapse experience. However, by opening eligibility to patients with more prior lines of therapy, the study may have expanded to include patients with more severe conditions and a longer duration of living with myeloma and therefore a different perspective. These patients may have come to terms with the severity of their condition and therefore minimized their symptom experience or treatment burden. In addition, our study population was composed only of white and Black/African American participants and as such may miss the experiences of patients from other ethnicities or racial groups. Although the absolute number of Black or African American participants in our study sample was small (*n* = 4), the proportion was higher than the general population (18.2% vs 13.4%) [26] and more closely approximates the ~ 20% MM case distribution observed in Black patients in the USA [27], as MM disproportionately affects Black patients [28]. Selection bias is possible due to the in-person interview design and outpatient sample. The study inclusion criteria focused on less severe cases, so the population was skewed towards less heavily treated patients (i.e., those with fewer relapses) who tend to be ambulatory (i.e., not hospitalized) with better functional status and more able to participate in the onsite in-person interviews conducted in this study. Finally, although we deliberately framed questions about symptoms in a way that separated symptoms experienced prior to RRMM diagnosis and symptoms experienced once treatment was initiated, it may have been difficult for participants to distinguish disease- or relapse-related symptoms from treatment-related symptoms. We cannot determine with certainty whether the patient-reported symptoms were in fact treatment-related, given a lack of physician corroboration, so the results reported herein represent a patient perspective on treatment-related symptoms.

## Conclusions

The data collected from this study provide valuable insight into treatment experience concepts that are relevant to patients with RRMM. The results can be used to improve our understanding of the symptoms, HRQOL impacts, and treatment burden themes that are most important to patients. Specifically, these findings suggest that factors related to treatment administration time are important to patients and should be considered in patient-provider communications in clinical contexts. The results can also be used to inform future research into treatment preferences among patients with RRMM.

## Supplementary Information

Below is the link to the electronic supplementary material.

Supplementary file1 (198 KB)

## Data Availability

Not applicable.
