# Supplementary material for: Patient perspectives on symptoms, health-related quality of life, and treatment experience associated with relapsed/refractory multiple myeloma
Source: Support Care Cancer. 2022 Apr 1;30(7):5859–69. doi: 10.1007/s00520-022-06979-7 (PMC9135799; doi:10.1007/s00520-022-06979-7)

Supplemental Material

Supplemental Figure 1. List of Concepts Used in Development of Semi-structured Qualitative Interview Guide

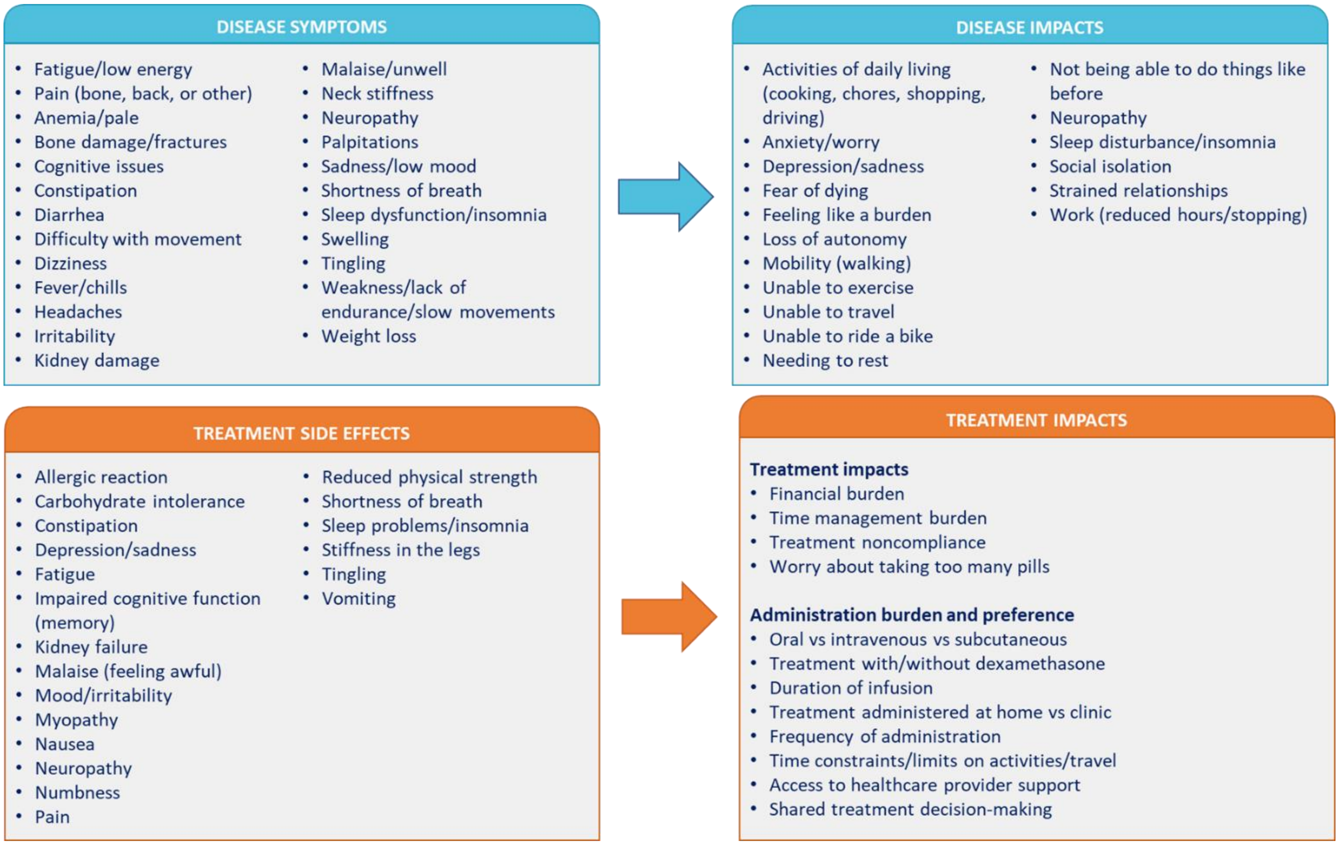

Supplement: Supplementary file 1 — Supplementary file1 (198 KB) [file 520_2022_6979_MOESM1_ESM.pdf]
